# Contemplate iliosacral screw in patients with developmental dysplasia of the hip

**DOI:** 10.1186/s13018-023-03606-x

**Published:** 2023-02-22

**Authors:** Ahmet Oztermeli, Nazım Karahan, Ahmet Aktan

**Affiliations:** 1 Gebze State Hospital, Osman Yılmaz, 1, İstanbul St. No:127, 41400 GebzeKocaeli, Turkey; 2 Corlu State Hospital, Orthopaedic and Traumatology, Zafer St, Bülent Ecevit Bvd. No:33, 59850 ÇorluTekirdağ, Turkey; 3 Taksim Gaziosmanpaşa Education Research Hospital, Osmanbey Bvd. 621 St, 34255 Gaziosmanpaşa, Istanbul, Turkey; 4 Gebze Fatih State Hospital, Orthopaedic and Traumatology, Zafer, Bülent Ecevit Blv. No:33, 59850 ÇorluTekirdağ, Turkey

**Keywords:** Iliosacral screw, Developmental dysplasia of the hip, Sacral dysplasia, Cross-sectional area, Hartofilakidis

## Abstract

**Objectives:**

Our aim in the study was evaluating sacroiliac morphology in patients with DDH and its possible effect on appropriate iliosacral screw fixation.

**Design:**

Retrospective cohort study.

**Setting:**

Level of evidence 3.

**Patients/participants:**

We evaluated the anteroposterior pelvis X-ray and pelvic CT scans of patients. We mainly divided the patients into two groups: DDH group (*n*:105) and control group (*n*:105).

**Intervention:**

The presence of the five qualitative characteristics of sacral dysplasia evaluated according to Route in both groups. The DDH group was divided into four subgroups according to the degree of hip dysplasia.

**Main outcome measurement:**

The cross-sectional area, length of the osseous corridor, coronal and vertical angulation evaluated in both groups.

**Results:**

The DDH group also exhibited a significantly higher S1 coronal and axial angulation, lower S1 cross-sectional area and S1 iliosacral screw length than the control group (*p*:0.033, *p*:0,002, *p*:0.006, *p*:0,019, respectively). According to the Rout classification, 9% were normal, 31% transient, 58% dysplastic in the DDH group. 45.7% were normal, 38% transient, 17% dysplastic in the control groups. These differences between the groups were statistically significant (*p* < 0.001). When the DDH groups were evaluated within themselves; no statistically significant difference was observed in S1 and S2 cross-sectional area, S1 and S2 maximum estimated iliosacral screw length, S1 and S2 axial and coronal angles assessment.

**Conclusion:**

Sacral dysplasia was more common, narrower and more angled osseous canal for the iliosacral screw was found in the DDH group. There was no relation between the degree of hip dysplasia and sacrum morphology in the DDH group. Thus, we suggest the surgeons be aware of iatrogenic injury even in constrained dysplastic hips.

## Introduction

Developmental dysplasia of the hip (DDH) is a condition with a progressive dysplastic deformity of femoral head and acetabulum [1, 2]. DDH could lead to an axial skeletal system pathology; leg length discrepancy, insufficient abductor muscle strength that can cause lumbar lordosis and also scoliosis [3, 4]. Dysplastic changes affect the entire pelvis, not just the acetabulum [5].Routt et al. classified the upper sacral segment morphology as normal, transitional, and dysplastic, according to the radiological characteristics. Six qualitative characteristics are determined for the dysmorphic sacrum [6]. Narrow and angled osseous corridor in the first sacral segment is found in patients with sacral dysplasia [7, 8]. Depending on gender and ethnicity, even in the normal population sacral dysplasia can be found up to 41% in the literature [8, 9].

It is important to evaluate these anatomical differences for pelvic surgery. In patients with sacroiliac joint dislocations, fracture dislocations or sacrum fractures, fixation of iliosacral screw percutaneously is applied with long screws which crosses the midline of sacrum [10, 11]. Sacral dysplastic changes can mislead the surgeon and iatrogenic injuries may occur constantly [9, 10, 12]. Also, sacral morphological changes could be contraindication for sacroiliac screw fixation [13].

Developmental dysplasia of the hip has different pelvic morphology such as greater pelvic incidence and pelvic tilt and also, sacral slope changes are seen in patients with DDH [14, 15]. In a study evaluating direct radiographs, it is shown that the degree of hip dysplasia affects the degree of pelvic morphological changes but there is no study to evaluate the degree of hip dysplasia and sacrum morphology with computer tomography (CT) [16]. Sacral slope is one of the parameters to evaluate sacral dysplasia so there could be a relation between DDH and sacral dysplasia.

Our aim in the study was evaluating sacroiliac morphology in patients with DDH and its possible effect on appropriate iliosacral screw fixation. Also the relation between the degree of dysplasia of the hip and sacral morphology was assessed.

## Materials and methods

### Patient selection

The anteroposterior pelvis radiography and pelvic CT scans of patients with adequate quality images and medical history available on the Picture Archiving Communication system (PACS) from January 2011 to September 2020 are evaluated. The patients' ages were between 20 and 86. Entire pelvis and the lowest rib-bearing vertebrae were included in the images. The study had a retrospective nature and got approval by the Local Ethics Committee.

Patients with center-edge angle less than 20°, Tonnis angle more than 13° on radiographs were defined as DDH [17]. According to this evaluation, we found 187 patients with DDH.

The exclusion criteria:

1. Patients with spine deformation such as spondylolisthesis, kyphosis, scoliosis, spinal stenosis or sacral agenesis.
2. Patients had teratologic hip dysplasia or cerebral palsy
3. Patients with pelvic tumor, history of pelvic trauma or surgery
4. Skeletal immature patients
5. Patients with general joint laxity
6. Rheumatological disease such as ankylosing spondylitis which had a possible effect on the spine and pelvis.
7. Patients did not have adequate preoperative computer tomography (CT) scans in the PACS.

After exclusion criteria were applied 105 patients were selected for the study in the DDH group. We recruited 105 patients with center-edge angle more than 20°, Tonnis angle less than 13° in the control group after the same exclusion criteria were applied. In the control group we evaluated only the right side.

The DDH group divided into four subgroups, patients with dysplastic changes but have a preserved hip containment were named as dysplastic group, patients with non-preserved hip containment were divided according to Hartofilakidis Classification. In type A group, the femoral head was in the acetabulum despite some subluxation with segmental deficiency of the upper wall. In type B group, the femoral head formed a false acetabulum above the true acetabulum, but the false acetabulum was not completely separated from the true acetabulum. In type C group, the femoral heads were not connected to the true acetabulum [18].

The DDH group and the control group were divided into 3 subgroups as Routt et al. suggested. Images containing 5 qualitative characters were evaluated as sacral dysplasia which is an upper sacral segment not recessed in the pelvis, the presence of mammillary processes, an acute alar slope, a residual disc between the first and second sacral segments and noncircular upper sacral neural foramina [6]. The patients did not have any of the 5 qualitative characters described as the normal group. The patients have all of the 5 qualitative characters described as the dysplastic group. The patients have less than 5 qualitative characters described as the transient group.

The cross-sectional area, length of the osseous corridor, coronal and vertical angulation were compared between the groups.

### Data collection

Patient’s demographic data; body mass index (BMI), age, gender and indication of CT scan were recorded retrospectively. Intraobserver reproducibility was determined by calculating intraclass correlation coefficients (ICCs) of 30 randomly chosen patients. This evaluation was made twice 10 days apart.

**Fig. 1:**
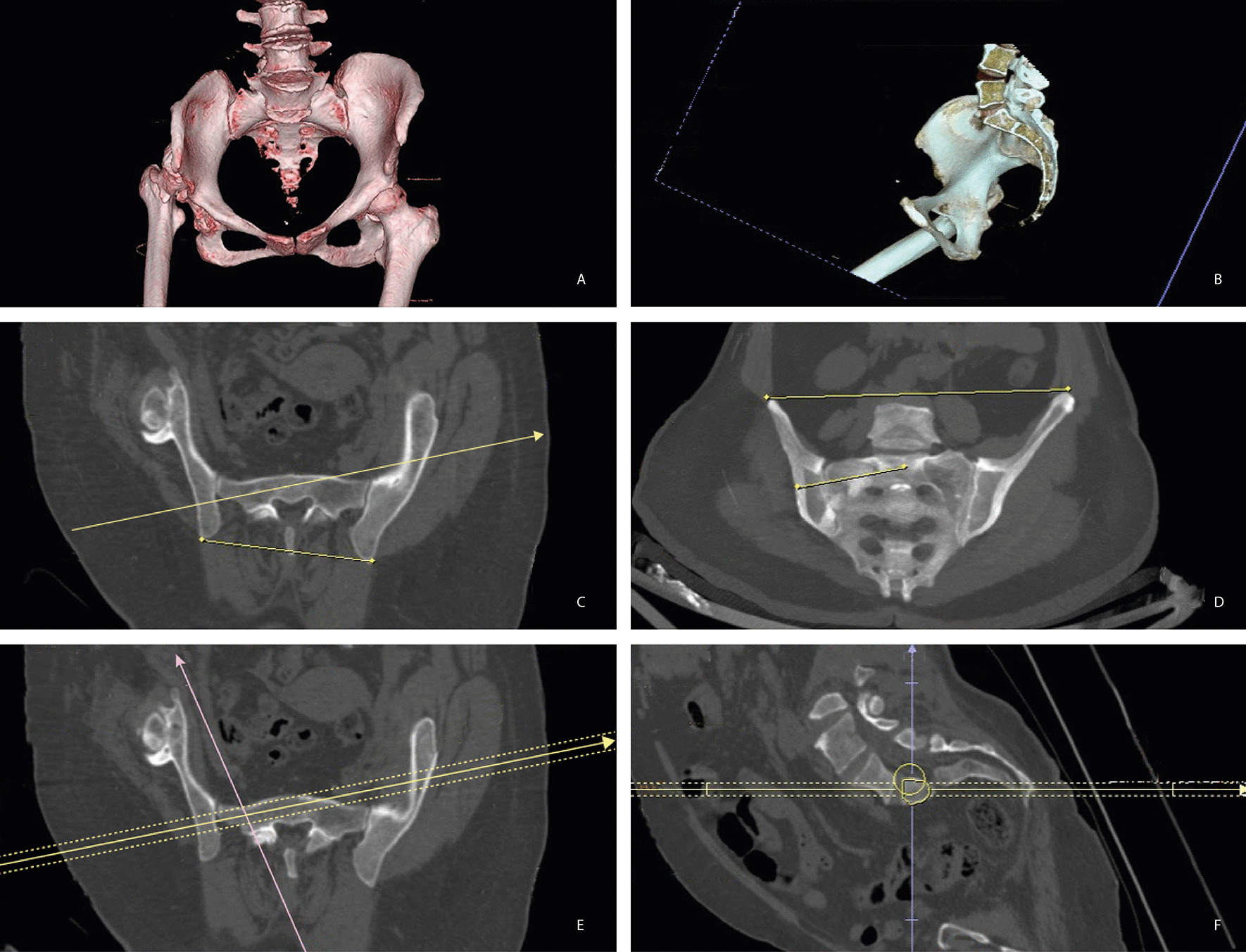
3D pelvic CT reconstruction images were analyzed in a 55-year-old patient with right unilateral DDH. **A** Bilateral sacral dysmorphism, DDHon the right side are seen. **B** More than half of the S1 body located above the iliac cortical density on the right. **C** ve **D** axial and coronal reformats made perpendicular to the S1 and S2 sacral osseous corridors. Axial and coronal angulations of the first and second sacral segments were measured. **E** The maximum length of a 10 mm diameter osseous corridor measured. **F** Sagittal computed tomography image showing the cross-sectional area of the safe zone

High resolution CT with 16 sections (Somatom Emotion; Siemens Healthcare, Germany) was used for the study and a 0.625 mm slice interval while the angle was at 0.1° with the precision of 0.1 mm in length was taken. Reconstruction of the images was made by PACS (Infinitt, Korea). The cross-sectional areas and short width of the safe zones were measured as described by Kim et al.’s study; the oblique sagittal images on multiplanar reformation images was used in the same screen that was on contiguous slices perpendicular to the axis of the sacral osseous corridor on axial reformats [10]. The sagittal projection angle was evaluated in true pelvic outlet view, because of the different spinopelvic geometry of each patient. Based on pelvic outlet view, coronal angulation was measured using a line drawn perpendicular to the axis of the osseous corridor and a line connecting the top of the iliac crests. Similarly, the sagittal projection angle was evaluated in true pelvic inlet view for each patient. Axial angulation was measured using a line drawn perpendicular to the axis of the osseous corridor and a line connecting the posterior iliac spines [9] (Fig. 1).

The CT scans of patients were analyzed by a single radiologist with at least 5 years of experience in musculoskeletal imaging who is blinded to the study.

## Statistical analysis

Statistical analysis was performed using the SPSS version 25 software (IBM Corporation, Armonk, New York, United States). A statistical power analysis was performed for sample size estimation. the projected sample size needed with this effect size (GPower 3.1) is approximately *N* = 88 for this simplest between group comparison. Thus, our proposed sample size of 105 will be more than adequate for the main objective of this study. Data were analyzed using descriptive statistics (mean, standard deviation, median, frequency, percentage, minimum, and maximum). The normal distribution of the data was evaluated using the Shapiro–Wilk test. Variance homogeneity was assessed using the Levene test. The intraobserver reproducibility was determined using intraclass correlation coefficients (ICCs). One-way ANOVA post hoc Tukey test was performed to compare descriptive statistical data (mean, standard deviation, median, frequency, ratio, minimum, and maximum) and quantitative data among the three groups with a normal distribution. The Student’s T-test was used for the comparison of the two groups that showed a normal distribution. The Mann–Whitney U test was used for the comparison of the two groups that showed a normal distribution. Pearson’s chi-square test was used to compare qualitative data. A *p*-value < 0.05 was considered statistically significant.

## Results

No significant difference was observed between the groups according to age, gender and BMI (Table 1).

**Table 1:** The distribution of descriptive characteristics of two groups

Groups	DDH (*n* = 105)	Control (*n* = 105)	*p*-value
*Gender*			
Female	39(37.1%)	69(65.7%)	0.586^A^
Male	66(62.8%)	36(34.2%)	
BMI	23.9 ± 2.92	24.6 ± 3.09	0.641^B^
*Side*			
Right	42(40%)		
Left	39(37.1%)		
Bilateral	24(22.8%)		
*Age (years)*			
Mean ± SD	51.3 ± 19.08	51.3 ± 14.76	0.745^B^
Range	20–86	20–85	

^A^Pearson Chi-square test

^B^Mann–Whitney U

S1 coronal and axial angulation were statistically higher and S1 cross-sectional area and S1 iliosacral screw length were statistically lower in the DDH group compared to control group (*p*:0.033, *p*:0.002, *p*:0.006, *p*:0,019, respectively). No significant difference was observed in S2 coronal and axial angulation, S2 cross-sectional area and S2 iliosacral screw length between the groups. No statistically significant difference was observed in S1 and S2 cross-sectional area, S1 and S2 maximum estimated iliosacral screw length, S1 and S2 axial and coronal angles assessment by gender, BMI, age in both groups (*p* > 0.05) (Table 2).

**Table 2:** Quantitative measures according to DDH groups and control groups

	DDH group	Control group	*p
S1 Minimum cross-sectional area (mm^2^)	371.34 ± 57.34	414.7 ± 81.93	0.006
S1 Maximum iliosacral screw length (mm)	81.39 ± 8.16 (63.4–102.4)	87.03 ± 8.09 (71.9–104.1)	0.019
S1 Axial angulation (degree)	22.02 ± 7.82(8.10–39.5)	15.98 ± 5.53 (4.2–28.5)	0.002
S1 Coronal angulation (degree)	18.02 ± 6.83 (9.1–30.2)	15.39 ± 4.82 (5.6–24.4)	0.033
S2 Minimum cross-sectional area (mm^2^)	181.3 ± 54.18	191.7 ± 56.22	0.082
S2 Maximum iliosacral screw length (mm)	54.60 ± 7.06(41.4–79.6)	57.48 ± 9.07(40.40–74.70)	0.264
S2 Axial angulation (degree)	8.54 ± 4.23(1.2–16)	6.93 ± 3.49 (1.10–16.9)	0.108
S2 Coronal angulation (degree)	7.86 ± 3.5 (1.60–15.5)	7.08 ± 3.8(1.30–12.70)	0.646

*Student *t* test

According to the Rout classification, 9% were normal, 31% transient, 58% dysplastic in the DDH group. 45.7% were normal, 38% transient, 17% dysplastic in the control groups. These differences between the groups were statistically significant (*p* < 0.001) (Table 3).

**Table 3:** Comparison of qualitative data according to the Rout classification

Routt classifications	DDH group(*n*:129)	Control group(*n*:105)	*p
Normal	12(9%)	48 (45.7%)	< 0.001
Transient	40(31%)	40 (38%)	
Displastic	76(58%)	17 (16.1%)	

*Pearson Chi-square test

When the DDH groups were evaluated within themselves; no statistically significant difference was observed in S1 and S2 cross-sectional area, S1 and S2 maximum estimated iliosacral screw length, S1 and S2 axial and coronal angles assessment (Table 4).

**Table 4:** Comparison of measurements in the DDH group according to the degree of dysplasia

	Dysplastic group (*n*:55)	Type A (*n*:39)	Type B(*n*:21)	Type C(*n*:14)	P*
S1 Minimum cross-sectional area (mm^2^)	391.8 ± 95.5	361.7 ± 71	382 ± 47.73	365.1 ± 42.66	0.311
S1 Maximum iliosacral screw length (mm)	86.10 ± 7.21	83.12 ± 8.79	78.92 ± 8.11	84.40 ± 4.23	0.652
S1 Axial angulation (degree)	17.6 ± 6.81	23.46 ± 8.58	21.36 ± 7.63	19.7 ± 6.86	0.656
S1 Coronal angulation (degree)	16.11 ± 5.55	19.74 ± 4.12	17.52 ± 5.87	15.62 ± 6.63	0.332
S2 Minimum cross-sectional area (mm^2^)	197 ± 57.74	191.48 ± 59.2	193.07 ± 67.2	160 ± 35.5	0.498
S2 Maximum iliosacral screw length (mm)	76.7 ± 8.68	78.89 ± 9.13	75.4 ± 8.01	79.9 ± 8.48	0.620
S2 Axial angulation (degree)	7.68 ± 5.32	8.37 ± 4.43	9.42 ± 3.44	6.05 ± 6.08	0.441
S2 Coronal angulation (degree)	7.46 ± 2.74	7.88 ± 2.78	7.75 ± 4.19	8.15 ± 6.88	0.378

*One way Anova Turkey test

An ICC value of 0.9 was considered excellent, and values between 0.8 and 0.9 were considered good [17, 18]. The intraobserver ICCs were 0.83 for the S1 Minimal cross-sectional area, 0.84 for the S1 maximum iliosacral screw length, 0.86 for the S1 axial angulation, 0.85 for the S1 coronal angulation, 0.85 for the S2 Minimal cross-sectional area, 0.82 for the S2 maximum iliosacral screw length, 0.85 for the S2 axial angulation, 0.84 for the S2 coronal angulation.

## Discussion

The prominent finding in our study is that sacral dysplasia is more common in the DDH group than the control group. The second important finding is that the degree of hip dysplasia was not related with cross-sectional area, iliosacral screw length, axial and coronal angles in the DDH group. Another important finding is that narrower and more angled osseous canal was found in the DDH group.

Increased proximal femoral antiversion, shortened femoral neck, decreased femoral intramedullary canal, and oval-shaped acetabulum are the findings in patients with DDH, and the femoral head also migrates anteriorly and superiorly in the acetabulum [19, 20]. Increased lumbar lordosis, scoliosis, and knee joint valgus deformity appear in hip dislocation cases [3, 21]. This causes coronal malalignment in the lower extremities in DDH patients [22]. Pelvic incidence and sacral slope were different in patients with DDH compared to the normal population [14]. Coronal malalignment was found to be directly proportional to the degree of dislocation in DDH [23]. We expected more dysplastic changes in the sacrum as the degree of dislocation increased in patients with DDH, but we could not find a relationship between them.

The results in the literature evaluating the iliosacral screw length, cross-sectional area, axial and coronal angles according to gender were found to be variable. Some studies showed that the cross-sectional area, iliosacral screw length, and coronal and axial angle were lesser in women than in men [24, 25]. However, there is also a study showing that these parameters are not related to gender [26]. In our study, there was no relationship between these parameters and gender.

The mean axial angle for S1 was found 19.27 by Hasenboehler et al. [24]. Kaiser et al. [9] found the mean axial angle; 11 ± 10.5 and the mean coronal angle; 22.6 ± 11.1 for S1 and found that coronal and axial angulations were higher in patients with sacral dysplastic changes. Gardner et al. [8] found the mean axial angulation; 4.2 ± 3.60 and the mean coronal angulation; 20.5 ± 6.3 for the normal sacrum, and the mean axial angulation; 14.9 ± 0.9.0, and the coronal angulation; 30.3 ± 4.6 of the dysplastic sacrum. In our study, sacral dysplasia was more common in the DDH group, and axial and coronal angulations were found to be higher than in the control group. The mean axial and coronal angles in the control group were similar to the literature.

Screw diameter and number have an effect on the length of the iliosacral screw. Posterolateral-anteromedial direction is preferred for long screw placement [27, 28]. 6.3, 7 and 8 mm screws are commonly used and it is essential to leave a safe bone distance around the screw when selecting diameters. There are many studies evaluating the effects of differences in screw numbers and screw diameters [8, 29, 30]. In our study, we measured vertical and posterior placements for 8 mm screws. Since most of our patients were dysplastic, the cross-sectional area was narrow, so we did not prefer the use of multiple screws. To keep the bone stock adequate, we measured using a thickness of 10 mm by adding an additional 2 mm to the screw diameter.

Rout et al. classified the hips without any of the dysplastic findings as normal, those with all these findings as dysplastic, and those with some of these findings but not all as transient [6]. These 6 qualitative findings were associated with dysmorphic sacrum, but there is a study showing that the tongue-in groove finding is not a reliable marker [9]. Therefore, we did not accept the tongue-in groove finding as a parameter associated with sacrum dysplasia in our study. Routt et al. preferred to evaluate sacrum dysplasia with radiographic outlet images in their cadaver study [6]. However, we preferred to use computer tomography instead of conventional radiography, since sacral dysmorphism may also result from differences in the radiographic image used to evaluate sacral dysplasia [5].

Radiography was preferred for evaluate in hip dysplasia due to certain advantages, such as lower cost and quick evaluation. Ogata et al. [17] modified the Wiberg center-edge angle because, in some hips the classic center-edge angle was not reliable. And also, more intraobserver and interobserver variability was observed in modified center-edge angle than the classical Wiberg center-edge angle [31]. That's why we choose the modified Wiberg center-edge angle.

This study had limitations. First, according to the degree of hip dysplasia, the number of patients in the group is not equal. Second, all the patients were of Caucasian descent. It has been shown in the literature that ethnic differences have an impact on sacrum morphology. Further studies are needed for different ethnic groups. Another limitation is that it has been observed that 6 characteristic dysplastic characteristics have moderate interobserver reliability in order to classify according to Routt classification [9]. In our study, although strong intraobserver reliability was observed in the evaluation made with a single observer, it is thought that the evaluations made with different observers may affect the results. Another limitation was we included only the measurement of the right side of patients in control group. Mendel et al. showed that these measurement differs according to the side, however, no statistically significant difference was observed [25]. Thus, we thought that side selection did not affect the results. Last, this is a retrospective study so for definitive answer to the issue at hand, further comprehensive studies are needed.

## Conclusion

Sacral dysplasia was more common, narrower and more angled osseous canal for the iliosacral screw was found in the DDH group. There was no relation between the degree of hip dysplasia and sacrum morphology in the DDH group. Thus, we suggest the surgeons be aware of iatrogenic injury even in constrained dysplastic hips.

## Data Availability

The data that support the findings of this study are available on request from the corresponding author.

## Abbreviations

DDH: Developmental dysplasia of the hip
CT: Computer tomography
PACS: Picture Archiving Communication system
BMI: Body mass index
ICCs: Intraclass correlation coefficients

## References

[1] Clohisy JC, Dobson MA, Robison JF, Warth LC, Zheng J, Liu SS (2011). Radiographic structural abnormalities associated with premature, natural hip-joint failure. J Bone Jt Surg Am.

[2] van Bosse H, Wedge JH, Babyn P (2015). How are dysplastic hips different? A three-dimensional CT study. Clin Orthop Relat Res.

[3] Crowe JF, Mani VJ, Ranawat CS (1979). Total hip replacement in congenital dislocation and dysplasia of the hip. J Bone Jt Surg Am.

[4] Weinstein SL (1987). Natural history of congenital hip dislocation (CDH) and hip dysplasia. Clin Orthop Relat Res.

[5] Fujii M, Nakashima Y, Sato T, Akiyama M, Iwamoto Y (2011). Pelvic deformity influences acetabular version and coverage in hip dysplasia. Clin Orthop Relat Res.

[6] Routt ML, Simonian PT, Agnew SG, Mann FA (1996). Radiographic recognition of the sacral alar slope for optimal placement of iliosacral screws: a cadaveric and clinical study. J Orthop Trauma.

[7] Conflitti JM, Graves ML, Routt MLC (2010). Radiographic quantification and analysis of dysmorphic upper sacral osseous anatomy and associated iliosacral screw insertions. J Orthop Trauma.

[8] Gardner MJ, Morshed S, Nork SE, Ricci WM, Routt MLC (2010). Quantification of the upper and second sacral segment safe zones in normal and dysmorphic sacra. J Orthop Trauma.

[9] Kaiser SP, Gardner MJ, Liu J, Routt ML, Morshed S (2014). Anatomic determinants of sacral Dysmorphism and implications for safe Iliosacral screw placement. J Bone Jt Surg Am.

[10] Kim J-J, Jung C-Y, Eastman J, Oh H-K (2016). Measurement of optimal insertion angle for Iliosacral screw fixation using three-dimensional computed tomography scans. Clin Orthop Surg.

[11] Nork SE, Jones CB, Harding SP, Mirza SK, Routt MLC (2001). Percutaneous stabilization of U-shaped sacral fractures using iliosacral screws: technique and early results. J Orthop Trauma.

[12] Miller AN, Routt ML (2012). Variations in sacral morphology and implications for iliosacral screw fixation. J Am Acad Orthop Surg.

[13] Ilharreborde B, Breitel D, Lenoir T, Mosnier T, Skalli W, Guigui P (2009). Pelvic ring fractures internal fi xation: Iliosacral screws versus sacroiliac hinge fixation. Orthop Traumatol Surg Res.

[14] Imai N, Miyasaka D, Tsuchiya K, Suzuki H, Ito T, Minato I (2018). Evaluation of pelvic morphology in female patients with developmental dysplasia of the hip using three-dimensional computed tomography: a cross-sectional study. J Orthop Sci.

[15] Okuda T, Fujita T, Kaneuji A, Miaki K, Yasuda Y, Matsumoto T (2007). Stage-specific sagittal spinopelvic alignment changes in osteoarthritis of the hip secondary to developmental hip dysplasia. Spine.

[16] Li YM, Li JH, Li B, Wang JX, Chen YS (2016). The radiological research for pelvis asymmetry of unilateral developmental dysplasia of the hip in adult. Saudi Med J.

[17] Ogata S, Moriya H, Tsuchiya K, Akita T, Kamegaya M, Someya M (1990). Acetabular cover in congenital dislocation of the hip. J Bone Jt Surg Br.

[18] Hartofilakidis G, Lampropoulou-Adamidou K (2016). Lessons learned from study of congenital hip disease in adults. World J Orthop.

[19] Wells J, Nepple JJ, Crook K, Ross JR, Bedi A, Schoenecker P (2017). Femoral morphology in the dysplastic hip: three-dimensional characterizations with CT. Clin Orthop Relat Res.

[20] Nepple JJ, Wells J, Ross JR, Bedi A, Schoenecker PL, Clohisy JC (2017). Three patterns of acetabular deficiency are common in young adult patients with acetabular dysplasia. Clin Orthop Relat Res.

[21] Li Q, Kadhim M, Zhang L, Cheng X, Zhao Q, Li L (2014). Knee joint changes in patients with neglected developmental hip dysplasia: a prospective case-control study. Knee.

[22] Kilicarslan K, Yalcin N, Cicek H, Cila E, Yildirim H (2012). What happens at the adjacent knee joint after total hip arthroplasty of Crowe type III and IV dysplastic hips?. J Arthroplasty.

[23] Kocabiyik A, Misir A, Kizkapan TB, Yildiz KI, Kaygusuz MA, Alpay Y, Ezici A (2017). Changes in hip, knee, and ankle coronal alignments after total hip arthroplasty with transverse femoral shortening osteotomy for unilateral Crowe type IV developmental dysplasia of the hip. J Arthroplasty.

[24] Hasenboehler EA, Stahel PF, Williams A, Smith WR, Newman JT, Symonds DL (2011). Prevalence of sacral dysmorphia in a prospective trauma population: implications for a “safe” surgical corridor for sacro-iliac screw placement. Patient Saf Surg.

[25] Mendel T, Noser H, Kuervers J, Goehre F, Hofmann GO, Radetzki F (2013). The influence of sacral morphology on the existence of secure S1 and S2 transverse bone corridors for iliosacroiliac screw fixation. Injury.

[26] Balling H (2020). Gender-associated differences in sacral morphology do not affect feasibility rates of transsacral screw insertion. Radioanatomic investigation based on pelvic cross-sectional imaging of 200 individuals. Spine.

[27] Wu LP, Li YK, Li YM, Zhang YQ, Zhong SZ (2009). Variable morphology of the sacrum in a Chinese population. Clin Anat.

[28] Day CS, Prayson MJ, Shuler TE, Towers J, Gruen GS (2000). Transsacral versus modified pelvic landmarks for percutaneous iliosacral screw placement: a computed tomographic analysis and cadaveric study. Am J Orthop.

[29] Ziran BH, Wasan AD, Marks DM, Olson SA, Chapman MW (2007). Fluoroscopic imaging guides of the posterior pelvis pertaining to iliosacral screw placement. J Trauma.

[30] Moed BR, Geer BL (2006). S2 iliosacral screw fixation for disruptions of the posterior pelvic ring: a report of 49 cases. J Orthop Trauma.

[31] Omeroglu H, Biçimoglu A, Aguş H, Tümer Y (2002). Measurement of center-edge angle in developmental dysplasia of the hip: a comparison of two methods in patients under 20 years of age. Skeletal Radiol.

[32] Karahan N, Oztermeli A, Aktan A et al. Evaluation of Sacrum Morphology in Patient with Developmental Dysplasia of the Hip for Iliosacral Screw Fixation, 04 March 2021, PREPRINT (Version 1). Available at Research Square [10.21203/rs.3.rs-274930/v1].

